# Supplementary material for: Defects controlled hole doping and multivalley transport in SnSe single crystals
Source: Nat Commun. 2018 Jan 3;9:47. doi: 10.1038/s41467-017-02566-1 (PMC5752673; doi:10.1038/s41467-017-02566-1)
Supplement: Supplementary file 1 — Supplementary Information [file 41467_2017_2566_MOESM1_ESM.pdf]

Supplementary Information

Supplementary Note 1. Methods for crystal syntheses

As discussed in the 'Methods' section of the main text, we have synthesized 12 batches of SnSe single crystals. Three techniques are used to grow SnSe single crystals, *i.e.*, self-flux (SF), Bridgeman (BR), and physical vapor deposition (PVD). A tubular furnace and two two-temperature zone furnaces are used for the self-flux, Bridgeman and PVD methods, respectively. As shown in Supplementary Figure **1**, for the self-flux method, the growth flux is placed in the tabular furnace, and SnSe crystals nucleate and grow during the cooling stage; for the Bridgeman method, the tilted tube end holding the flux is placed in the low temperature zone (1223 K), while the other end is placed in the high temperature zone (1273 K), in order to form a temperature gradient for the growth; for the PVD method, we first synthesized SnSe crystal by the fast-cooling SF method. The as-grown single crystals with $\sim 1\%$ $SnSe_2$ were then ground thoroughly and sealed again. In the final step of PVD, the quartz tube was placed in a two-temperature zone furnace with a temperature gradient about 50 K over 12 cm. The detailed heating and cooling processes are summarized in Supplementary Table **1**.

Supplementary Note 2. Powder XRD and the determination of crystal orientations

The high quality of our single crystals is confirmed by the XRD results of polycrystal samples ground from high-quality single crystals. The results show the characteristic Bragg diffraction peaks perfectly matching the No. 62 space group (Supplementary Figure **2**). Along the a-axis, SnSe single crystals tend to form large edges of 45 degrees aligned to the b (zigzag)- and c (armchair)-axes. To determine the exact crystallographic orientations, which are critical for understanding the anisotropic transport in SnSe and a fully unfolding of the first Brillouin zone by ARPES, we cut the samples along the two 45-degree axes relative to the natural edges (as shown in the Inset of Supplementary Figure **2**). Then, the two cross-sections of the rectangular samples are characterized by XRD. As shown in Supplementary Figure **3**, for the a-c plane, the Bragg peaks only exhibit the ($mk0$)-sets of Miller indexes

(the upper panel), while the a-b plane displays the ($m0l$) indexes (the lower panel). The appearance of the m index is mainly due to the roughness of the cut cross-sections, which are typically slanted surfaces of a few degrees.

Supplementary Note 3. $SnSe_2$ micro-domains in SnSe crystals

$SnSe_2$ micro-domains are first identified during the micro-exfoliation of SnSe single crystals, aiming for the device fabrications of few-layer SnSe flakes. We discover that the thinnest flakes with a few nanometers in thickness always show distinct Raman peaks from thick films and the bulk SnSe. As shown in Supplementary Figure **4**c, the Raman peaks at $110\,cm^{-1}$ and $189\,cm^{-1}$ for few-layer samples ($< 10\,nm$) represent the E_g and A_g^1 phonon modes of bulk $SnSe_2$ [1], in stark contrast to the four fingerprinting phonon modes of A_g^1, B_g^3 , A_g^2, and A_g^3 for the Pnma phase (Supplementary Figure **4**b and **5**). Such a difference can not be attributed to the sensitivity of few-layer SnSe thin films to the air, which has been a serious issue for BP. Indeed, we have carried out the exfoliation of SnSe in a glove box with argon atmosphere, followed by *in-situ* spin coating of 500 nm-thick PMMA to protect SnSe thin films from air exposure. Polymer protected samples yield exactly the same Raman results, indicating that the thinnest flakes are $SnSe_2$ rather than SnSe. It is further found that these $SnSe_2$ micro-domains show high optical contrast on the SnSe bulk in both the bright-field and dark-field modes. As shown in Supplementary Figure **6**, SnSe has highly reflective surface, which is silvery under optical microscope like graphite, while $SnSe_2$ micro-domains are reddish with layer-dependent optical contrast. The existence of the $SnSe_2$ phase is also confirmed by AFM, which show that the interlayer distance of $SnSe_2$ is 0.60(7) nm, significantly larger than the step height of 0.53(3) nm for SnSe. The energy level alignment diagram in Figure 1d of the main text is based on scanning Kelvin probe microscopy (SKPM) and DFT. Supplementary Figure **7** shows the SKPM determined work functions of bare SnSe surface, compared with few-layer $SnSe_2$/SnSe heterostructure and bulk $SnSe_2$. The details has been discussed in the main text.

Supplementary Note 4. Carrier concentration determined by Hall measurements and Shubnikov-de Haas oscillation analysis

In Hall measurements, carrier concentration is derived by the single band model using the equation $n = -\frac{1}{R_{\mathrm{H}}e}$, where R_{H} is Hall coefficient extracted by the linear fitting of Hall resistivity below 1 T. Supplementary Figure **8**a shows the typical B-dependent Hall resistivity at various temperatures for SF3-SnSe. Note that quantum oscillations are superimposed on the linear Hall signals at high magnetic fields. After subtracting the B-dependent linear background of Supplementary Figure **8**a, we obtained the corresponding quantum oscillation curves in Hall resistivity. As shown in Supplementary Figure **8**b, at the same T, the quantum oscillations in Hall are essentially a π phase shift of the SdH oscillations in MR. The T-dependent hole concentrations of SF1- and SF3-SnSe are shown in Supplementary Figure **9** and the inset of Figure 1f in the main text. Below 50 K, there is surprising increase in the carrier density, which is consistent with the T-dependent SdH oscillations (Supplementary Figure **10**), which reveals reducing Fermi surface as T increases from the helium temperature.

We have also estimated carrier concentrations in different batches of SnSe using the SdH oscillation frequencies, which represent the FS cross-sections perpendicular to $\mathbf{B}$. By assuming spherical FSs, carrier concentrations can be deduced by the equation $n = \frac{2k_{\mathrm{SdH}}^3}{3\pi^2}$. Note that a factor of two is multiplied here to take into account the multi-valley transport in p-SnSe. Despite the over simplified FS assumption, hole concentrations in different batches of p-SnSe agree with the Hall measurements, as summarised in Table 1 in the main text. Alternatively, we can compare the second derivatives of the SdH oscillations at 1.5 K as a function of $1/B$. As shown in Supplementary Figure **11**, we can clearly see the shifts in the Landau levels for different metallic batches. This is mainly due to different hole doping levels in p-SnSe. Generally, higher hole doping suppresses SdH oscillations and pushes the same LL peak/valley to higher B. Such trend agrees well with the RT carrier concentrations measured by Hall effect.

Supplementary Note 5. Doping levels dependent Seebeck coefficients at room temperature.

For a single parabolic band in conventional doped semiconductors, one should observe $n^{-\frac{2}{3}}$ dependent Seebeck coefficient. Supplementary Figure **12** plots the Seebeck coefficients for different batches of SnSe single crystals with various doping levels at room temperature. It is clear that Seebeck coefficients of p-SnSe does not follow the $-2/3$ power law scaling as a function of doping, which is not surprising since SnSe belongs to a multi-valley band system.

Supplementary Note 6. Anisotropic MR in SnSe

We have systematically studied the effect of **I**-directions on the MR of a as grown BR1-SnSe sample, which shows 4 diagonal, two c (armchair)-, and two b (zigzag)- edges, respectively. Supplementary Figure **13**a shows the MR curves for different **I** setups, normalised by the zero field resistivity. Here the transverse magnetic field is fixed along the a-axis, and **I** adopts one of the nine setups, as illustrated in the inset of Supplementary Figure **13**a. For example, setup 1 corresponds to the configuration when **I** is applied between contact 1 and 9 (1'), while MR is measured between 3 and 7. Noticeably, when **I** is applied along the c-axis (armchair), regardless of the SdH oscillations, the MR curve shows non-saturating behaviour up to 14 T. By tilting **I** away from the c-axis, the low-filed weak localisation behaviour below 2 T are nearly identical. However, the conventional positive MR, corresponding to the cyclotron movement of charge carriers in **B**, become predominant above 2 T. Supplementary Figure **13**b exhibits the normalised MR of each setup at 14 T, which follows a sine-function dependence on θ, **I** and the c-axis. The twofold symmetry over the whole angle range ($0° \sim 360°$) reminds us of the robust in-plane dipole filed along the armchair direction, which probably plays an important role on the cyclotron movement of charge carriers.

Supplementary Note 7. ARPES supplemental information

We compared the Fermi surface contour plots for SF1-SnSe and SF3-SnSe in Supplementary Figure **14**. Supplementary Figure **14**a is the same as the 2D contour of $E_\mathrm{F} = 0$ eV in

Figure 2i. Supplementary Figure **14**b is the constant energy contour plot for $k_a - k_c$. Supplementary Figure **14**c shows the constant energy contour plot for $k_b - k_c$ of SF3, revealing a smaller Fermi surface compared to SF1-SnSe.

Supplementary Note 8. Effective mass of metallic SnSe

The T-dependent Landau level amplitudes of SF1- and SF3-SnSe with different configurations of $\mathbf{B}$ and $\mathbf{I}$ are fitted by the Lifshitz-Kosevich formula $\Delta R \propto \frac{14.69m^*T/Bm_e}{sinh(14.69m^*T/Bm_e)}$ to extract the effective mass m^* [2]. Supplementary Figure **15** shows the T-dependent MR, the residual MR after subtracting a smooth background, and the LK fitting results for SF1-SnSe with four different measurement configurations. The results are summarised in Supplementary Table **2**, agreeing well with the ARPES measurements, but drastically different from the DFT calculations for $\mathbf{B}||b$ and $\mathbf{I}||c$, or, $\mathrm{B}||c$ and $\mathbf{I}||b$. Supplementary Figure **16** shows the T-dependent MR and the residual MR of SF3-SnSe, with the corresponding LK-fit curves present in Figure 4e of the main text.

Supplementary Note 9. Weak localisation analysis

The NMR of SF1- and SF3-SnSe at low field below 2 T can be well explained by the quantum interference effect, *i.e.* weak localisation. At low T, the wave functions of charge carriers, backscattered by self-intersecting defect scattering loops, interfere with the time-reversed paths, leading to the increase of the probability of backscattering. Due to the quantum nature, this effect is very sensitive to temperature changes due to rapid decrement of quantum dephasing time τ_ϕ as T increases. In 3D metals or semiconductors, the dominant dephasing process is correlated to inelastic electron-phonon (e-p) scattering, which means $\tau_\phi \sim \tau_{ie-p}$. Typically, τ_ϕ inversely scales with temperature as T^{-p}, where $p = 2 \sim 4$, depending on the disorder intensity. For a quantitative analysis, we define an new parameter [3] $q_T l \sim \frac{k_B T l}{v_s \hbar}$, in which l and v_s are the mean free path and the average sound velocity (typically $2000 \sim 3000\,ms^{-1}$ for metals), respectively. In the clean (intermediate) regime with $q_T l \gg 1$, the scale factor is found to be $p = 2$, while it is $p = 3$ for the dirty limit, *i.e.* $q_T l \ll 1$. Using Fermi velocity $v_\mathrm{F} = 2.361 \times 10^5\,\mathrm{m\,s^{-1}}$ and $m^* = 0.258\,m_\mathrm{e}$ from ARPES measurements, we can further calculate l by the equation $l = \frac{\mu m^* v_\mathrm{F}}{e}$, in which the charge

carrier mobility μ is $700\,\mathrm{cm^2\,V^{-1}\,s^{-1}}$ from the Hall measurements calculated by $\mu = R_{\mathrm{H}}\sigma$. For SF1-SnSe, we get the parameter $q_T l$ to be between 1.19 and 2.38, suggesting intermediate disorder intensity.

For 3D materials, the behavior of NMR induced by suppressing the positive WL correction to resistivity can be modelled by the equation [4]:

$$\frac{\Delta\rho}{\rho^2} = -\alpha\frac{e^2}{2\pi^2\hbar}\sqrt{\frac{eB}{\hbar}}F(x), \tag{1}$$

in which $0 < \alpha < 1$ is a fitting coefficient representing the overall strength of the WL effect, B is the applied magnetic field, and $F(x)$ is the Hurwitz zeta function. Mathematically, $F(x)$ is given by,

$$F(x) = \sum_{n=0}^{\infty}[2(n+1+\frac{1}{x})^{0.5} - 2(n+\frac{1}{x})^{0.5} - 2(n+0.5+\frac{1}{x})^{-0.5}], \tag{2}$$

where $x = \frac{4eDB\tau_\phi}{\hbar}$, D is the electron diffusion constant defined as $\frac{\mu k_{\mathrm{B}}T}{e}$. In addition, there also exists positive MR in the quadratic form of γB^2 with the conventional origin. Thus, we have used three fitting parameters α, τ_ϕ and γ (shown in Supplementary Figure **17**) to simulate the MR characteristics of p-SnSe using the formula:

$$\mathrm{MR} = -\alpha\rho\frac{e^2}{2\pi^2\hbar}\sqrt{\frac{eB}{\hbar}}F(x) + \gamma B^2. \tag{3}$$

Supplementary Figure **17**d shows the derived T-dependent inelastic scattering time for SF1- and SF3-SnSe, which can be well fitted by $T^{-2-\delta}$, consistent with the previous analysis of intermediate disorder intensity in our system. According to the equation, $l_\phi = \sqrt{D\tau_\phi}$, we get the T-dependent phase coherence length for SF1 and SF3, plotted in Supplementary Figure **17**c. At 1.5K, $l_\phi \sim 100\,\mathrm{nm}$, much longer than the mean free path $l \sim 24.2\,\mathrm{nm}$, leading to the activation of quantum interference. The phase coherence length l_ϕ decreases as $T^{-0.5}$ from 1.5 K to 20 K. Above 20K, most backscattering paths are not phase coherent, and thus the NMR phenomena disappear. From Supplementary Figure **17**b, we can find that the quadratic contribution for **I** along c-axis is approaching zero above 5 K for SF1-SnSe, which makes the non-saturating NMR more conspicuous at elevated temperatures, as shown in Figure 3a of the main text. The physical origin of the non-saturating NMR behavior, which may closely related to the in-plane ferroelectric dipole field along the c-axis, may be answered by thickness-dependent study of SnSe thin-film devices.

Supplementary Note 10. Point dislocations and AFM images

Supplementary Figure **18** shows four typical AFM images obtained on randomly searched areas on freshly exfoliated SnSe surface (scanning locations are separated by 1 mm distance). We find that the dislocations are very common in p-SnSe single crystals. One detailed statistic result of 10 AFM images are summarised in Supplementary Table **3**. Supplementary Figure **19** plots the oxidation process of Sn rich surface in the vicinity of $SnSe_2$ micro-domains. The fresh exfoliated sample exhibits smooth surface. After air exposure for more than 30 hours, Sn rich surface is slowly oxidized with increasing roughness. On contrast, the bare SnSe surface does not show significant degradation during the experimental time scale of 72 hours. The Sn rich surface is created by the local phase segregation of $SnSe_2$ micro-domains, which leave excessive Sn accumulated at interlayer positions.

Supplementary Note 11. Tight-bonding model calculation results

Supplementary Figure **20**a and b plot the DFT calculations of constant-energy contours for $E_{\mathrm{B}} = 0.05$ eV and $E_{\mathrm{B}} = 0.075$ eV, showing pronounced differences from ARPES measurements (see Figure 2 and Supplementary Figure **14**) in the vicinity of the Fermi energy. To verify the reliability of our DFT results, we also calculate the band structure by the tight-bonding (TB) model. We use wannier90 program [5] to calculate the Wannier functions from a set of Bloch energy bands to get the TB results. As shown in Supplementary Figure **20**c, the contribution of density of states near E_{F} are predominantly from Se-4p, with low weight of Sn-5s and Sn-5p orbits. To get a more accurate results, we also take the Se-4s orbit into consideration. As shown in Supplementary Figure **20**d and **20**e, we find that the TB results reproduce the DFT results (Figure 2 in the main text) very well.

SUPPLEMENTARY REFERENCES

[1] Smith, A. J. Raman scattering studies of SnS_2 and $SnSe_2$. *Journal of Physics C: Solid State Physics* **10**, 1321 (1977).

[2] Shoenberg, D. *Magnetic Oscillations in Metals* (Cambridge University Press, 2009).

[3] Lin, J. J. & Bird, J. P. Topical review: Recent experimental studies of electron dephasing in metal and semiconductor mesoscopic structures. *Journal of Physics: Condensed Matter* **14**, R501 (2002).

[4] Kawabata, A. Theory of negative magnetoresistance i. : Application to heavily doped semiconductors. *Journal of the Physical Society of Japan* **49**, 628–637 (1980).

[5] Mostofi, A. A. *et al.* An updated version of wannier90: A tool for obtaining maximally-localised wannier functions. *Computer Physics Communications* **185**, 2309–2310 (2014).

SUPPLEMENTARY FIGURES

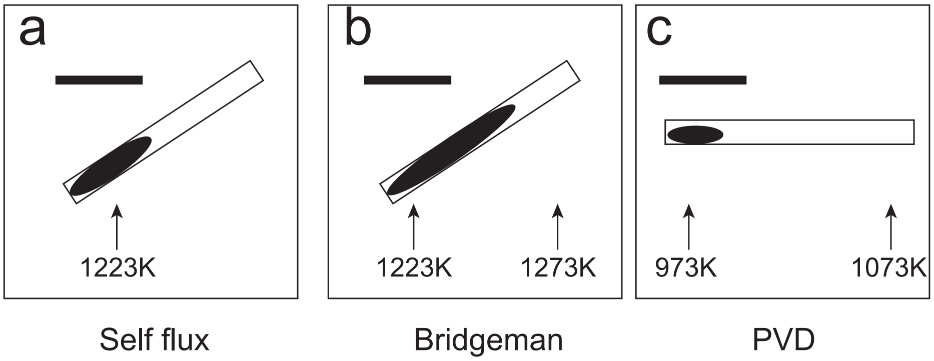

Supplementary Figure 1. **Sketches of three growth methods.** (**a**), self-flux, (**b**), Bridgeman and (**c**), PVD, respectively. The scale bar is 5 cm.

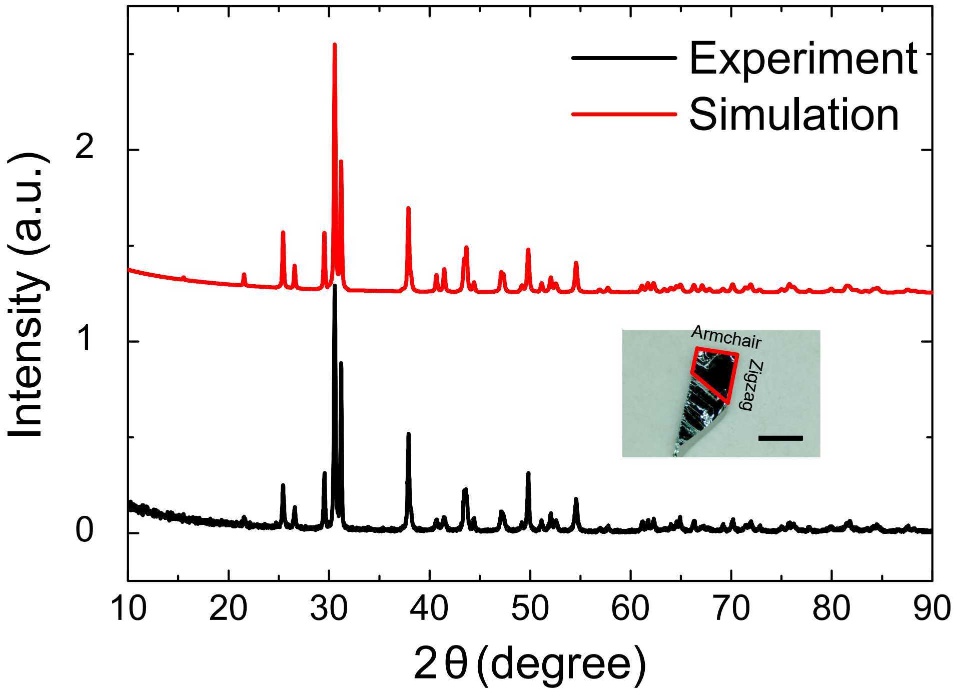

Supplementary Figure 2. **XRD patterns of ground polycrystals of SF1-SnSe and simulation.** The inset shows a typical sliced single crystal of SnSe, whose crystallographic axes are determined by XRD. The scale bar is 3 mm. Note that the large natural edge of the sample is along the diagonal of the c (armchair)- and b (zigzag)-axes.

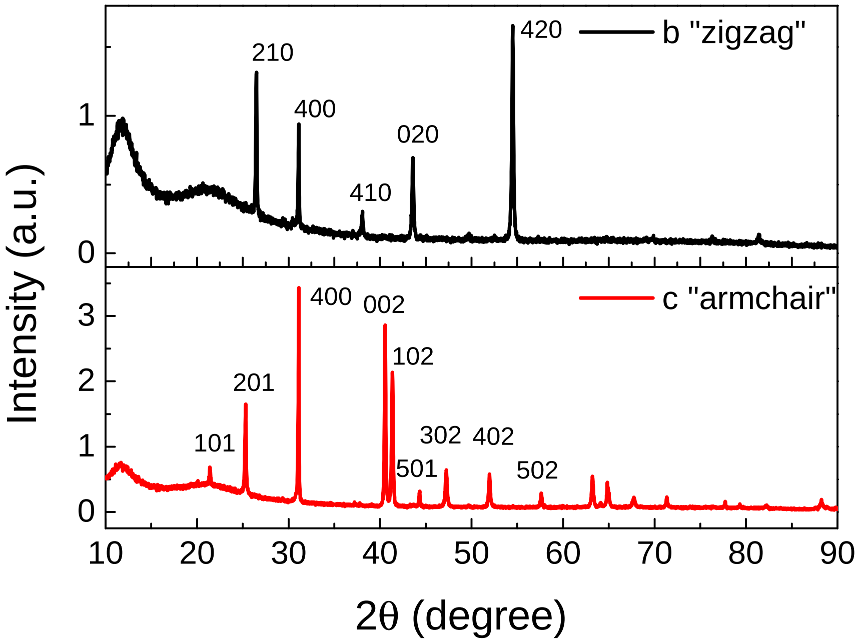

Supplementary Figure 3. **Edge orientations of SnSe single crystals determined by XRD.** The upper panel shows the ($ml0$) peaks, indicating the plane is along b-axis (zigzag). The lower panel shows the ($m0l$) peaks, indicating the plane is along c-axis (armchair).The appearance of m in the indexes is due to the slanted cutting of single crystals (typically a few degrees).

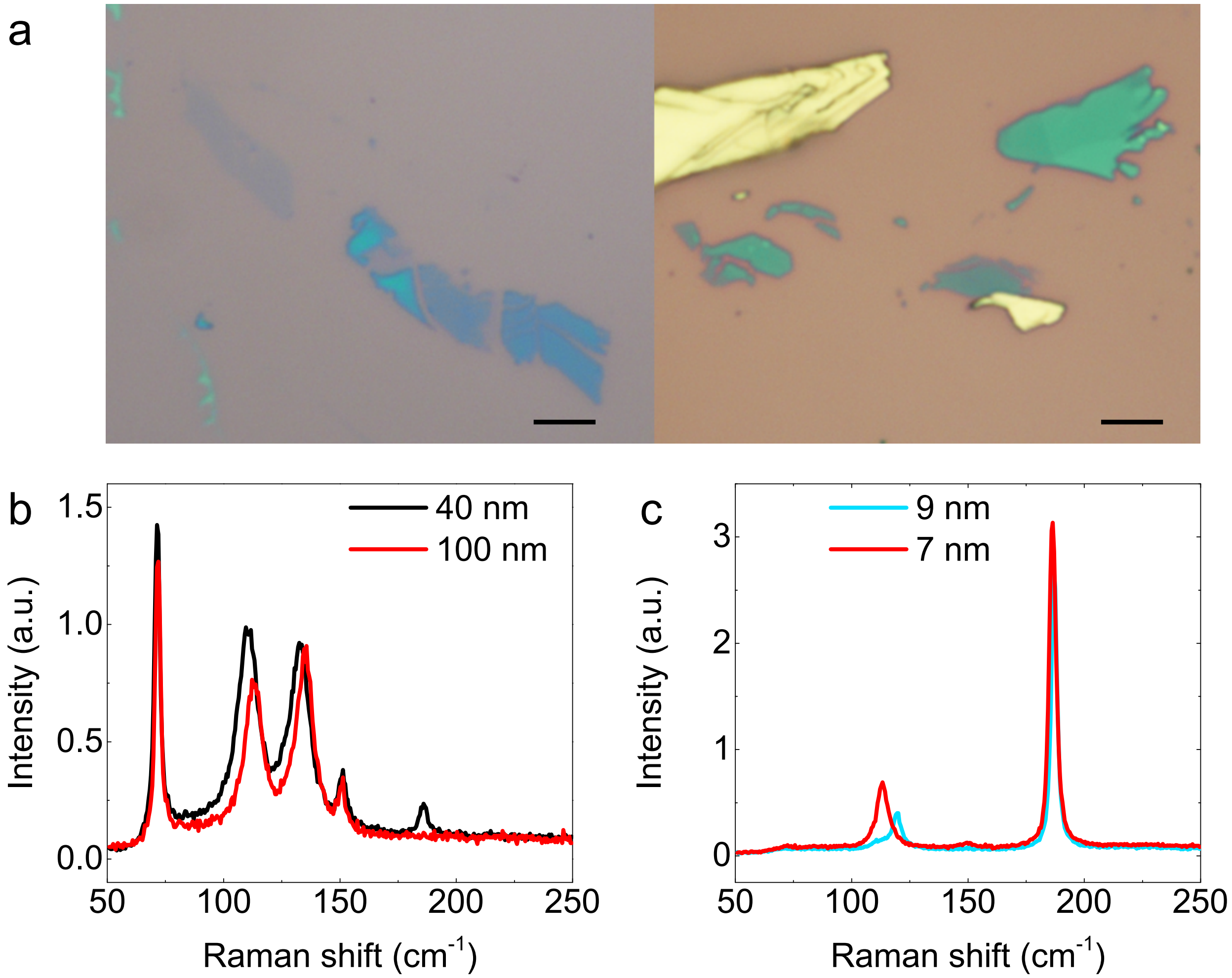

Supplementary Figure 4. **Optical images of exfoliated $SnSe_2$ flakes vs thick SnSe films.** (**a**) Left: Freshly exfoliated $SnSe_2$ few-layer flakes under 1000x optical microscope. Right: Greenish $SnSe_2$ and silvery thick films of SnSe, which is difficult to exfoliate due to point dislocations. The scale bar is $5\,\mu$m. (**b**) Raman spectra of silvery/greenish crystals, consistent with the results of bulk SnSe. (**c**) Raman spectra of the thinnest blueish flakes, showing Raman fingerprints of $SnSe_2$.

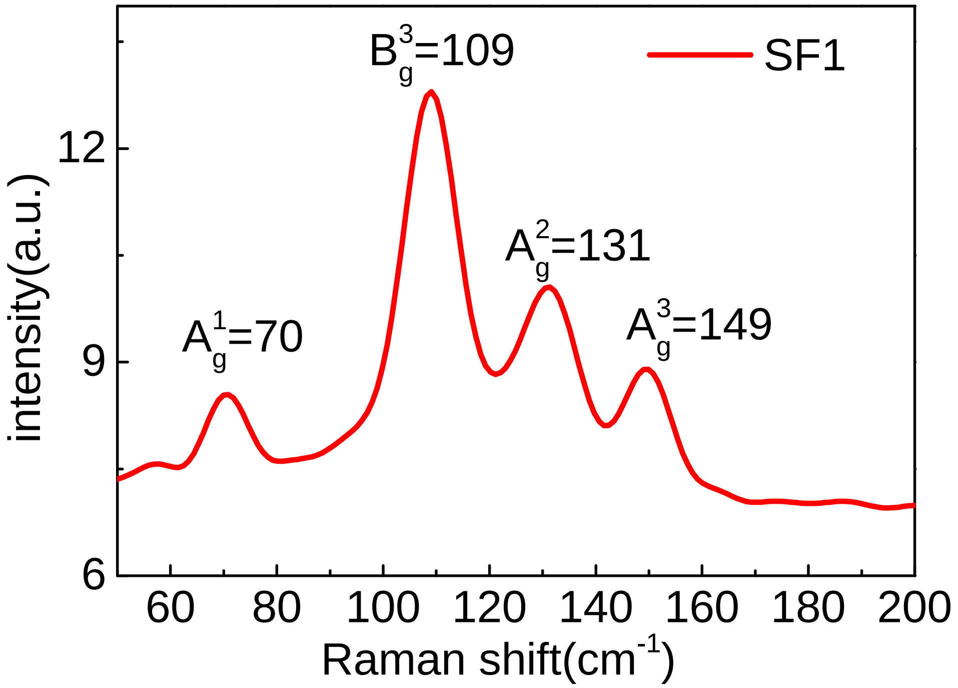

Supplementary Figure 5. **Raman spectroscopy of SnSe single crystal.** Raman spectrum of SnSe shows four fingerprinting phonon modes of A_g^1, B_g^3, A_g^2, and A_g^3 for the Pnma phase, which is consistent with previous reports.

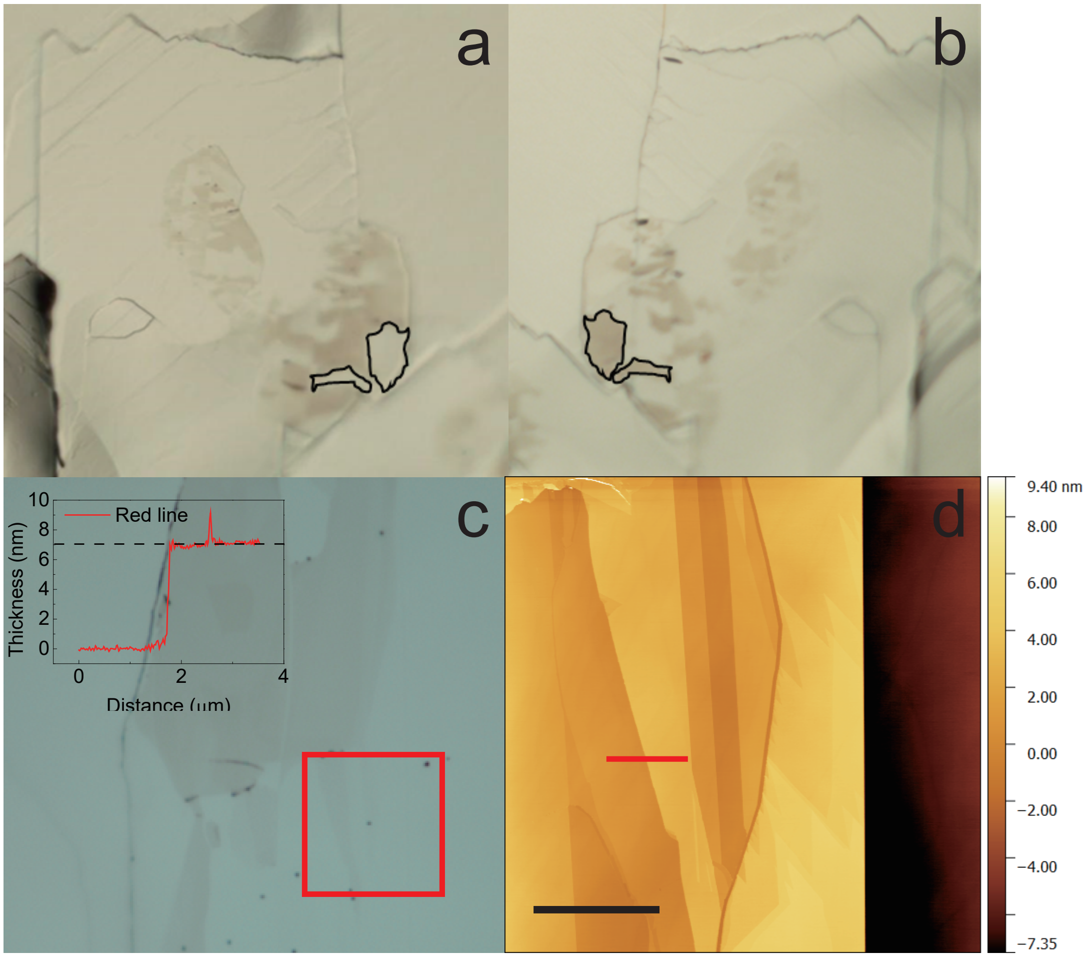

Supplementary Figure 6. **Optical contrast between $SnSe_2$ micro-domains and SnSe.** (**a**) The top surface of a freshly exfoliated SnSe single crystal (BR1), showing localized reddish $SnSe_2$ micro-domains. (**b**) The corresponding bottom surface of the detached top layers on the Scotch tape, suggesting that the micro-domains are randomly exfoliated. The black solid lines highlight $SnSe_2$ domains which are completely removed from the single crystal surface. (**c**) Layer-dependent optical contrast of $SnSe_2$ micro-domains on SnSe under 500x magnifications. (**d**) Nc-AFM image of a $SnSe_2$-SnSe mixed area in (**c**). The imaging locations are indicated by the red rectangle. By measuring the step height, we determined a thickness of 7 nm (10 monolayers) for the $SnSe_2$ domain. The scale bar is 4 μm.

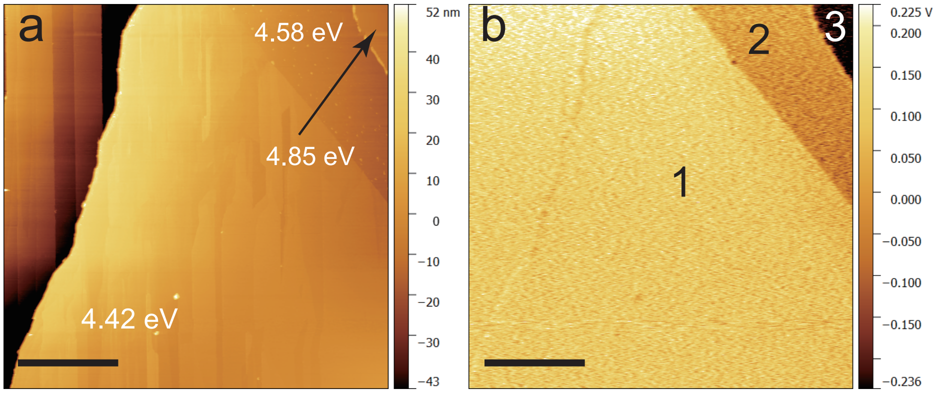

Supplementary Figure 7. **Topology (a) and SKPM (b) images of SnSe and SnSe/$SnSe_2$ heterostructure.** Area 1 with 4.42 eV is the bare SnSe surface. Area 2 with 4.58 eV work function is few-layer $SnSe_2$/SnSe heterostructure, while Area 3 with 4.85 eV is very thick $SnSe_2$ film, representing the bulk value. The scale bar is 4 μm.

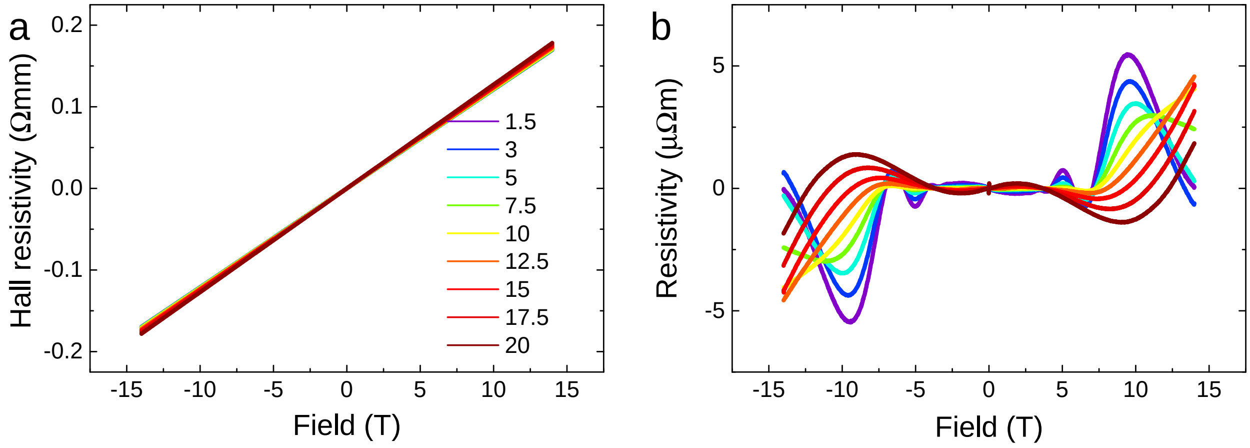

Supplementary Figure 8. **Hall resistivity of SF3-SnSe.** (**a**), B-dependent Hall resistivity at various temperatures and (**b**), the corresponding quantum oscillations after subtracting the linear background.The hole concentrations are extracted by the linear fitting of Hall resistivity below 1 T.

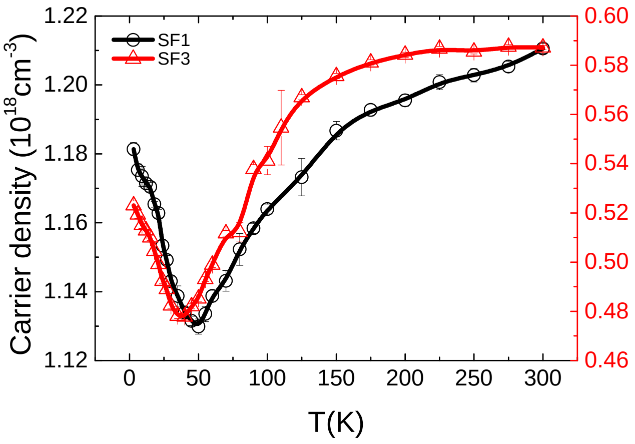

Supplementary Figure 9. **Hole carrier concentrations vs.** T **for SF1- and SF3-SnSe.** The error bars come from the slope error by linearly fitting the Hall resistivity.

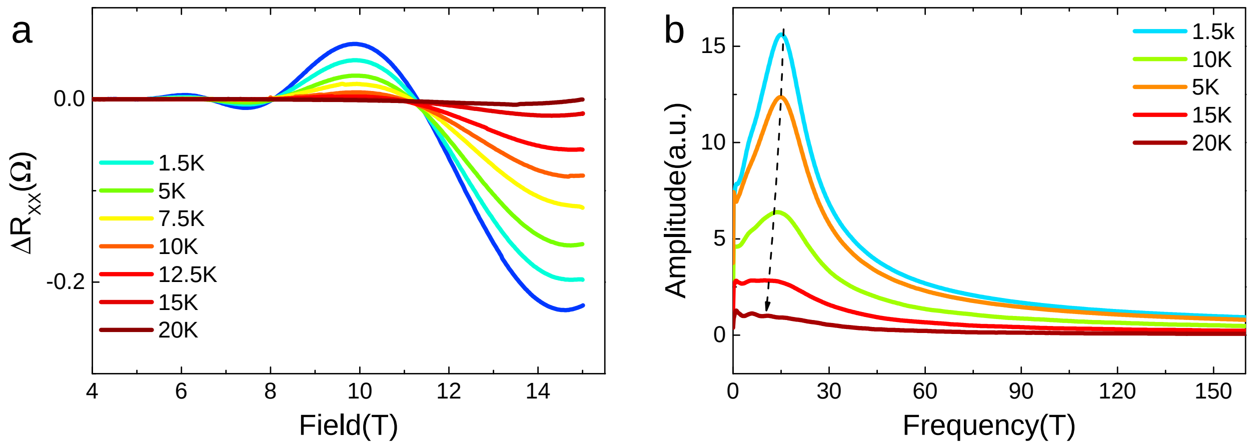

Supplementary Figure 10. **Shift of FFT frequencies.** (**a**), SdH oscillations vs. T for SF1-SnSe from 1.5 K to 20 K. (**b**), Using fast Fourier transform (FFT), we can clearly see systematic shifting in the peak positions of the FFT frequencies.

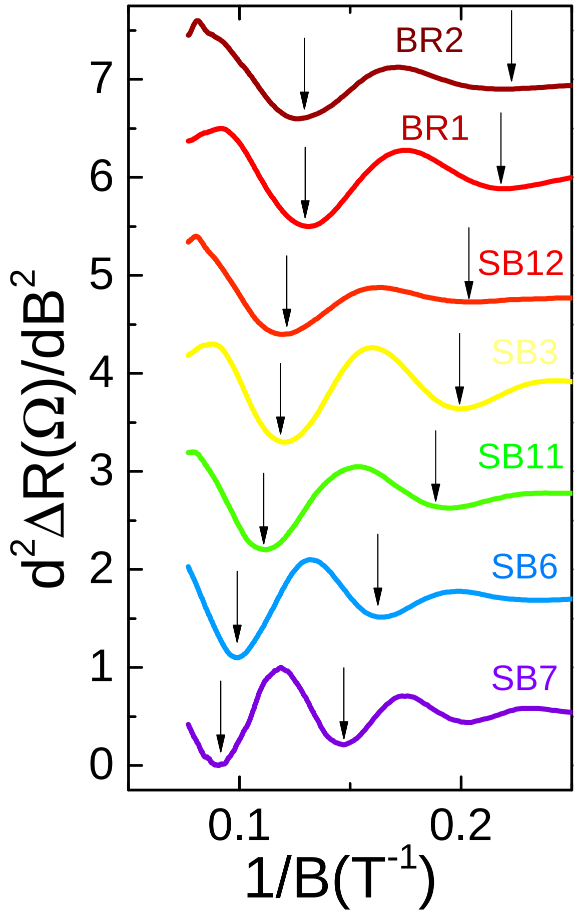

Supplementary Figure 11. Second derivative of the SdH oscillations for all metallic batches. The results are measured at 1.5 K and plotted as a function of reciprocal magnetic field, with the transverse field applied out of plane ($\mathbf{B}\|a$-axis).

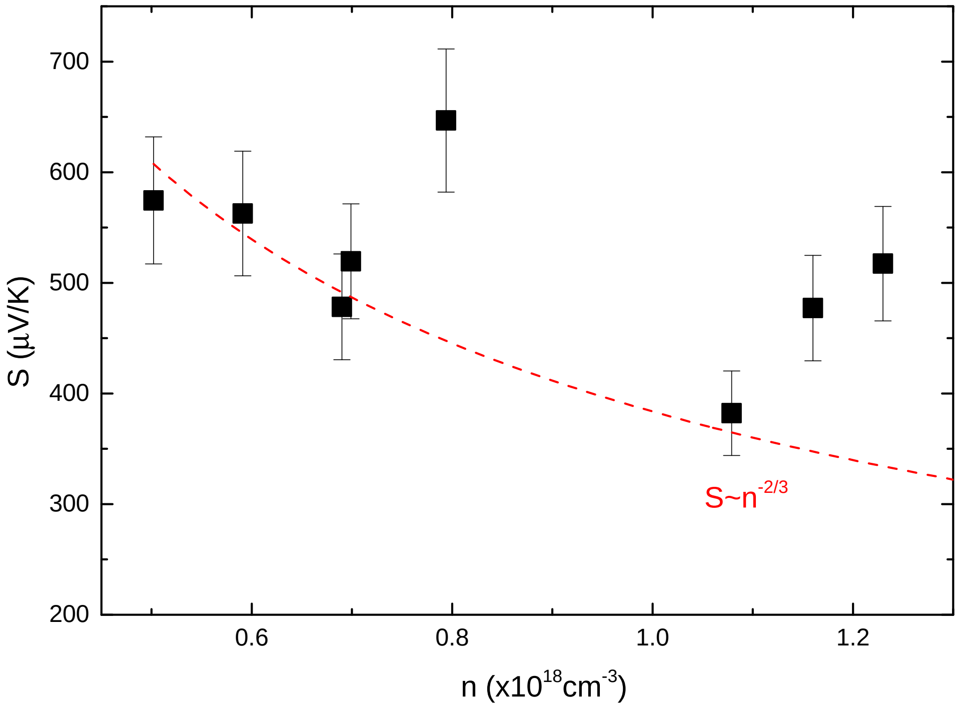

Supplementary Figure 12. **Seebeck coefficients for different batches of p-SnSe single crystals at room temperature.** The red dashed line the $n^{-\frac{2}{3}}$ scaling, which does not fit the experimental data very well. The error bars come from the systematical error of the setup for measuring the Seebeck coefficient, which typical has 20% error rate.

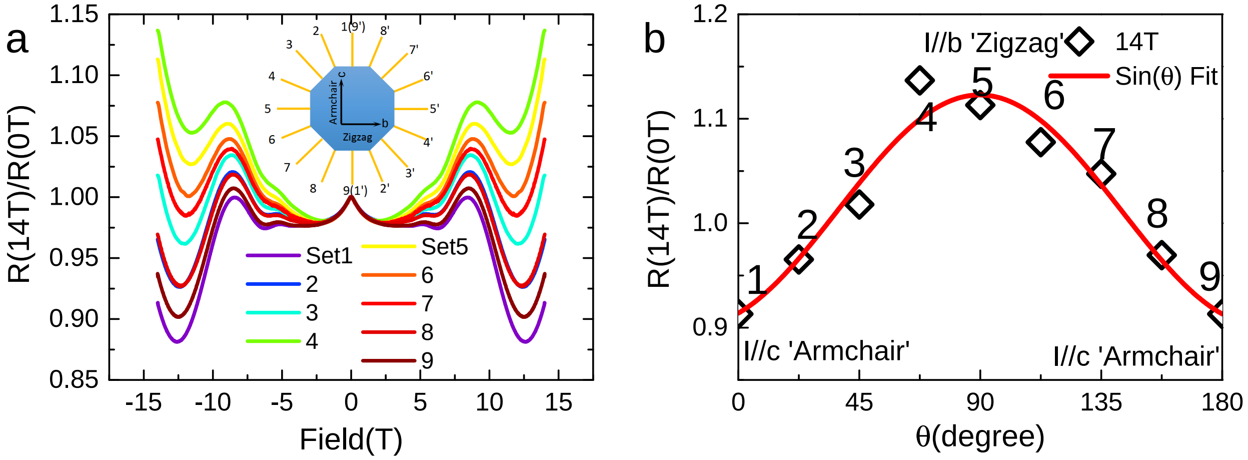

Supplementary Figure 13. **Anisotropic MR for I applied along various directions and B||a.** The MR curves are normalised by the zero field resistivity for better comparison. Note the exotic non-saturating behaviour up to 14 T for **I**||c-axis.

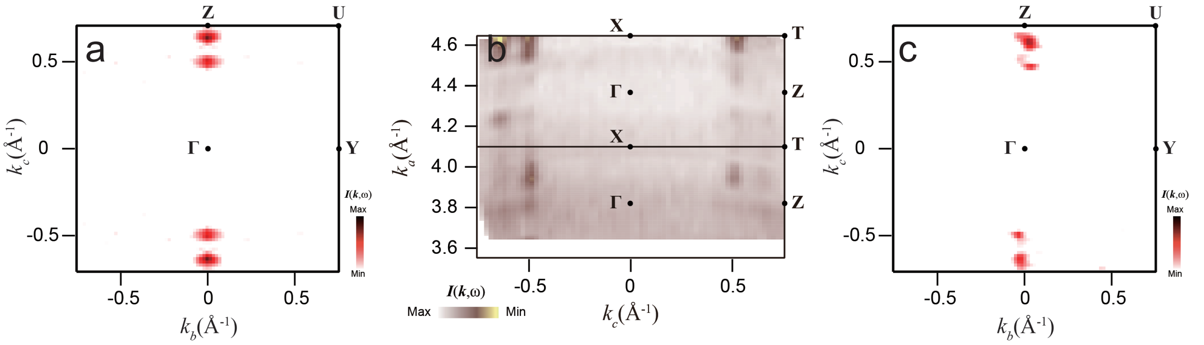

Supplementary Figure 14. **Counter plot of FSs of SF1 and SF3.** (**a**), $k_b - k_c$ and (**b**), $k_a - k_c$ planes for SF1, as well as (**c**), $k_b - k_c$ plane for SF3.

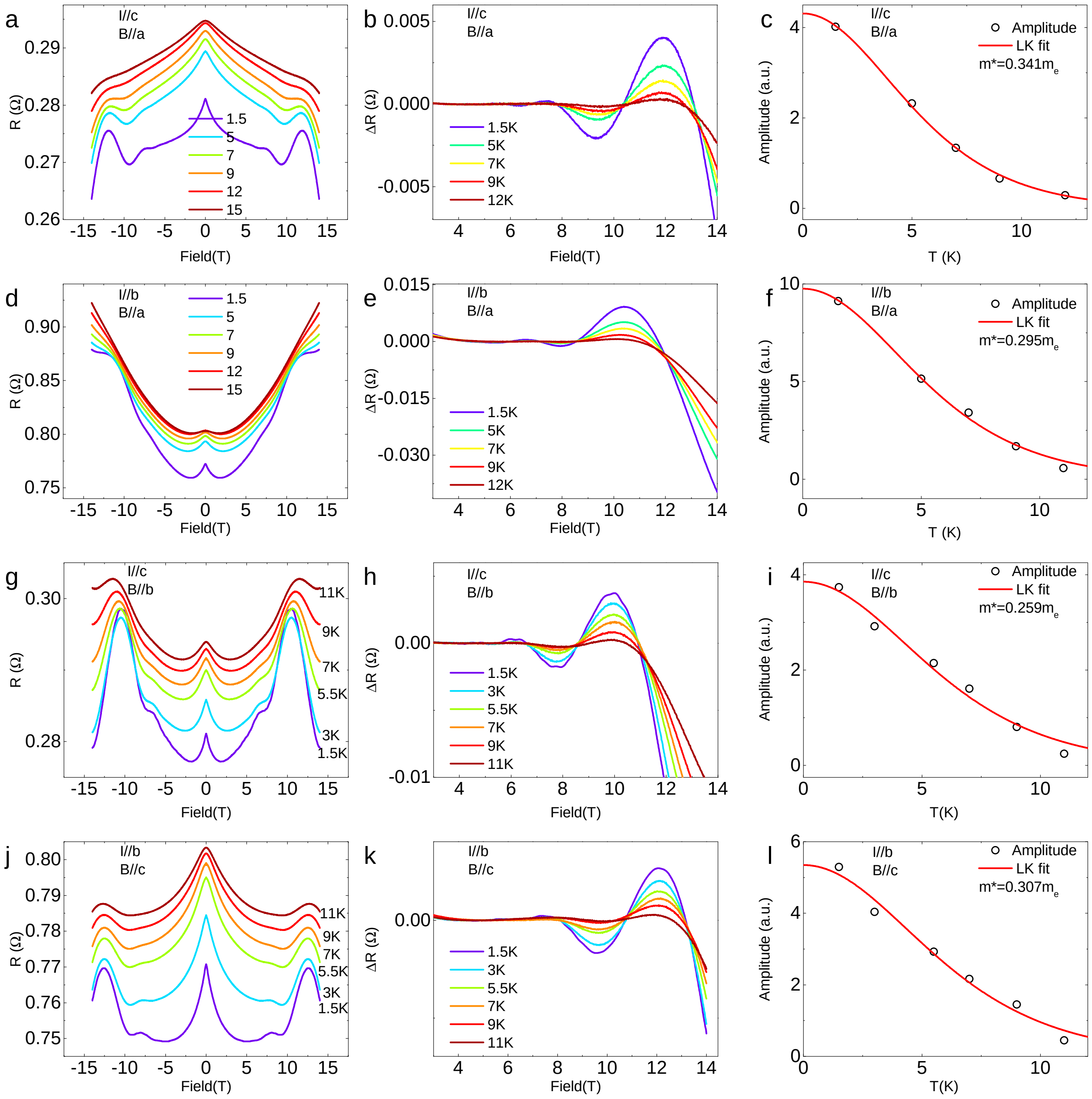

Supplementary Figure 15. Effective mass of SF1-SnSe for different configurations of I and B Left row: The T-dependent SdH oscillations of SF1-SnSe. Middle row: The residual MR after subtracting a smooth parabolic background. Right row: The LK-formula fitting of the T-dependent Landau level amplitudes (N=1). Four different **I** and **B** configurations have been analysed, namely, $\mathbf{I}\|c$ and $\mathbf{B}\|a$, $\mathbf{I}\|b$ and $\mathbf{B}\|a$, $\mathbf{I}\|c$ and $\mathbf{B}\|b$, and, $\mathbf{I}\|b$ and $\mathbf{B}\|c$.

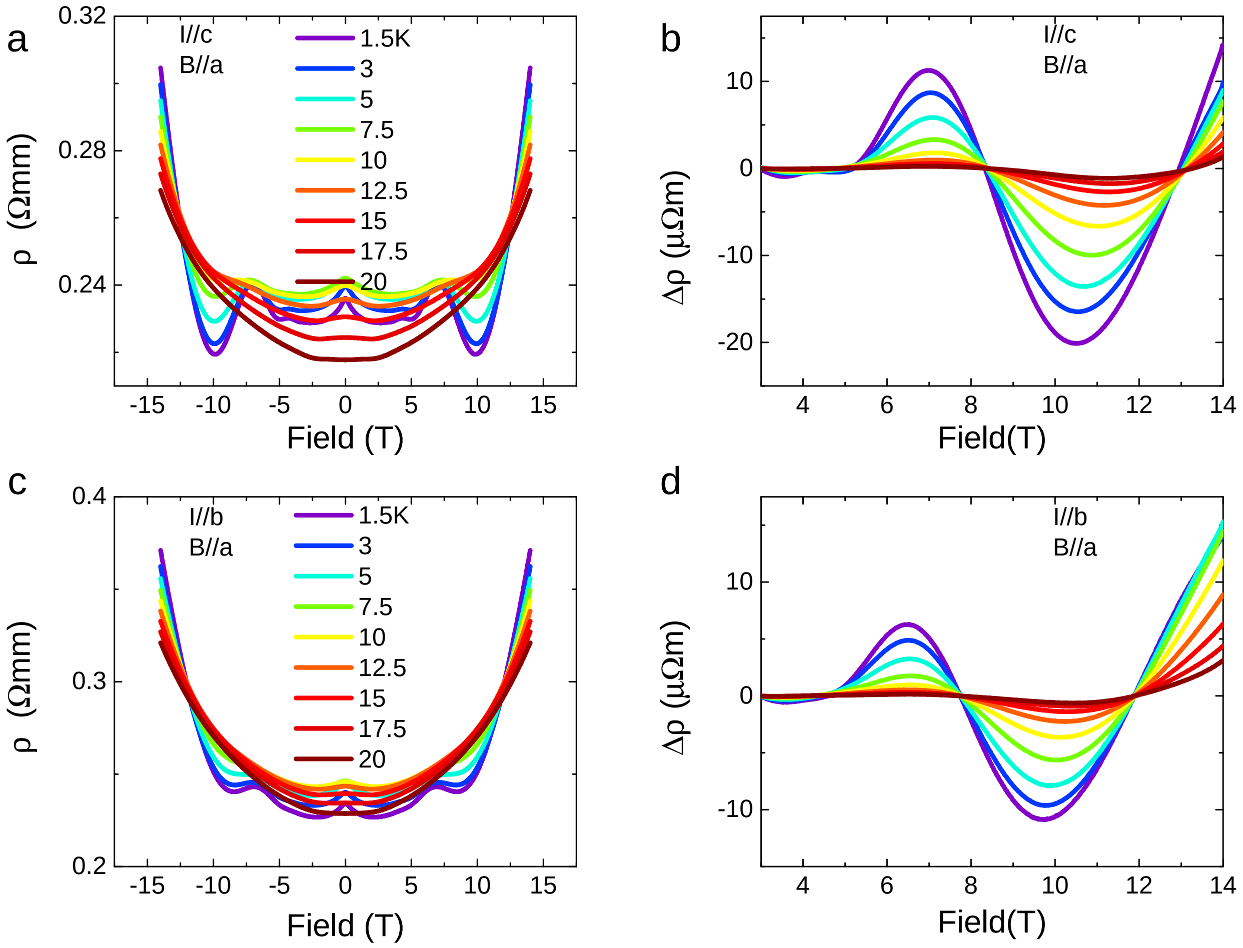

Supplementary Figure 16. **SdH oscillations of SF3-SnSe.** (**a**), (**b**), T-dependent SdH oscillations and residual MR after subtracting a smooth background for $\mathbf{B}\|a$, $\mathbf{I}\|c$ and (**c**), (**d**) for $\mathbf{B}\|a$, $\mathbf{I}\|b$.

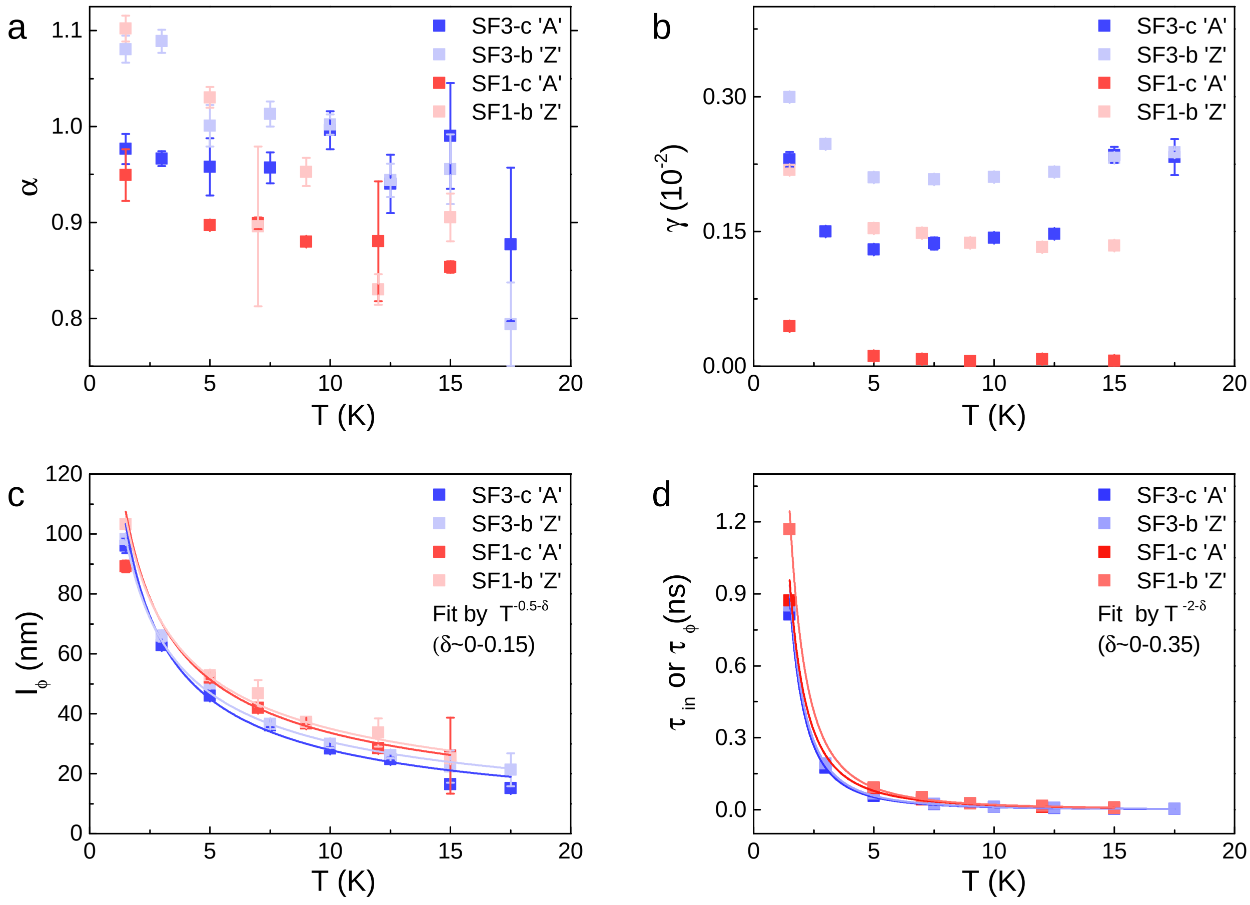

Supplementary Figure 17. **Parameters for simulating NMR of SF1- and SF3-SnSe.** (**a**), α is a fitting coefficient representing the overall strength of the WL effect. (**b**), γ is the quadratic MR from conventional contribution. (**c**), l_ϕ is phase coherence length. (**d**), τ_{in} is the inelastic electron-phonon scattering time, which is close to quantum dephasing time τ_ϕ in 3D semiconductors. The error bars come from the fitting errors for different parameters.

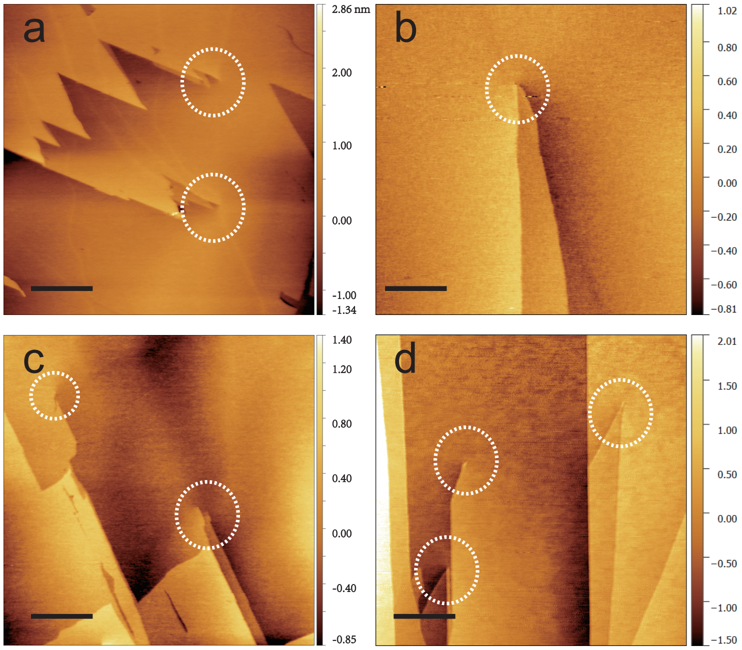

Supplementary Figure 18. **Point dislocations statistics of randomly chosen SnSe surface areas.** Point defects as highlighted by dashed circles are quite common in p-SnSe. The scale bars are 400 nm in (**a**) , and 600 nm in (**b**), (**c**) and (**d**).

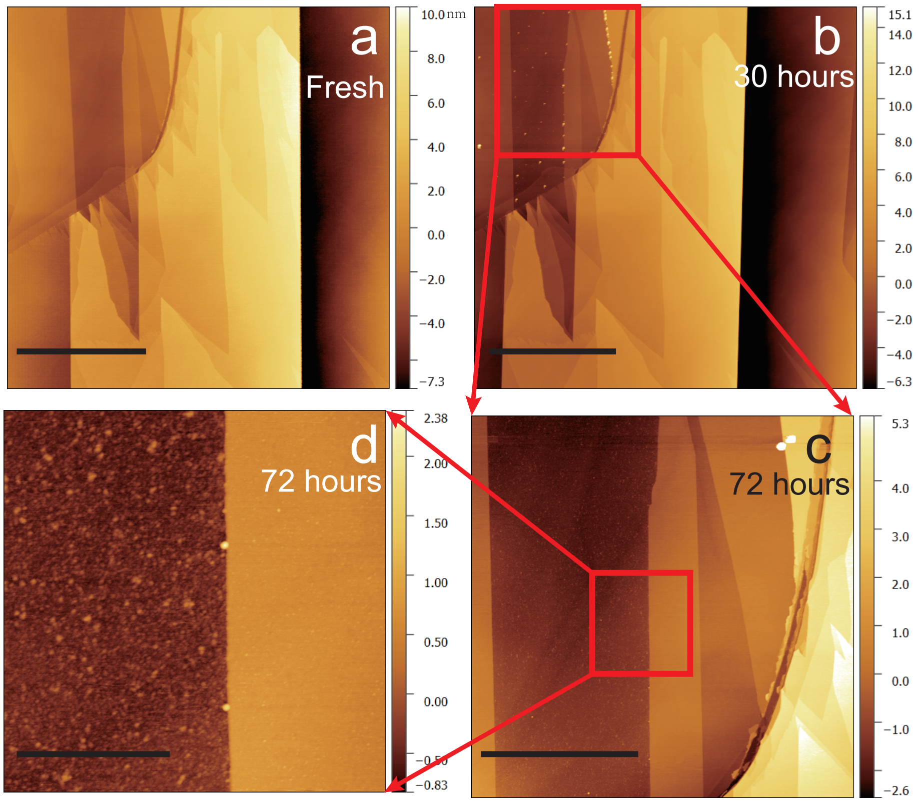

Supplementary Figure 19. **Sn rich surface in the vicinity of $SnSe_2$ micro-domains.** (**a**), fresh exfoliated surface of Sn rich areas. (**b**)∼(**d**), the Sn rich areas are gradually oxidized in air, while bare SnSe does not show significant degradation over 72 hours. The scale bars are 5 μm for (**a**) and (**b**), 2 μm for (**c**) and 400 nm for (**d**).

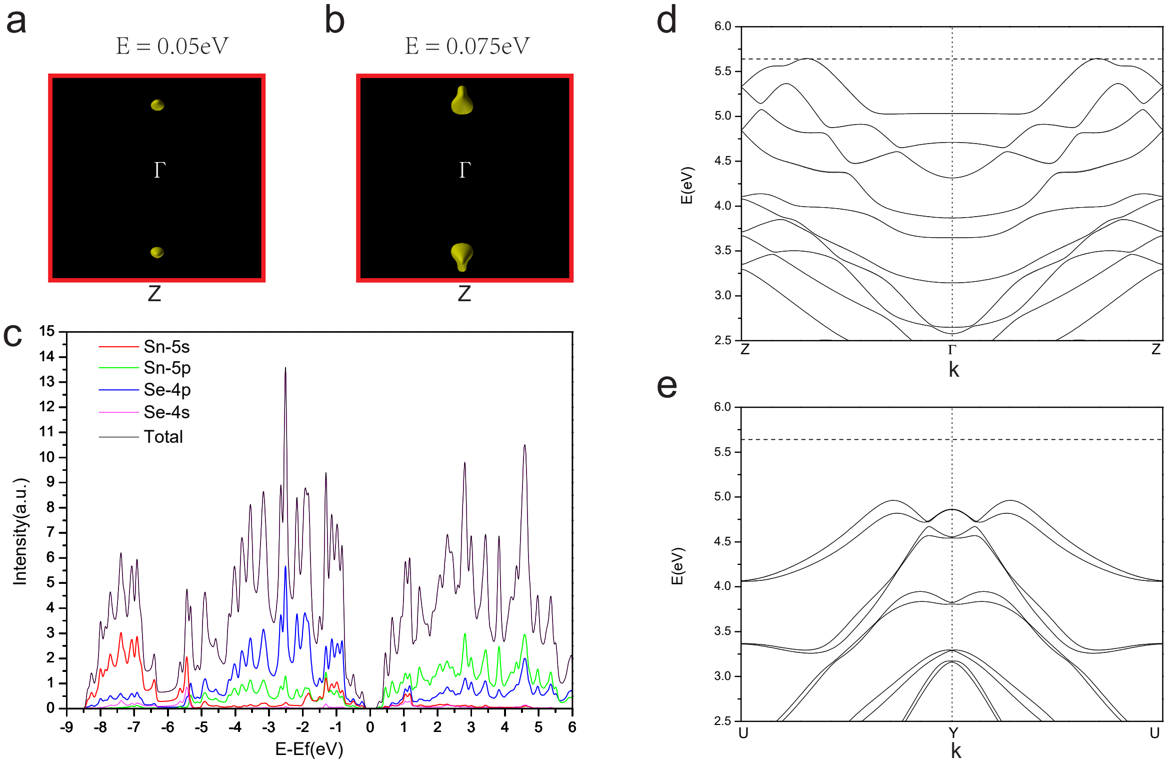

Supplementary Figure 20. **Tight-bonding calculations of the electronic structure of SnSe.**(**a**) and (**b**), the DFT calculations of constant-energy contours for $E_B = 0.05$ eV and $E_B = 0.075$ eV. (**c**), density of states near E_F by TB. (**d**) and (**e**), band calculations by TB.

SUPPLEMENTARY TABLES

Supplementary Table 1. Growth temperature curves for different batches of SnSe single crystals. The SF and BR methods have three main steps of “Heating up to the maximum growth temperature”, “Persistent time”, and “Flux cooling”, respectively.

Samples	Heating up to 1223K (mins)	Persistent time at 1223K (mins)	Flux cooling rate (mins)
SF1	600	1500	Fast cooling (1 day to 673 K)
SF7	600	1500	Fast cooling
SF6	600	1500	Fast cooling
SF3	600	3000	4000 to 673K, then natural
SF5	600	3000	4000 to 673K, then natural
SF8	600	6000	9999 to 673K, then natural
SF12	600 (Se:Sn=0.95)	7000	Fast cooling
SF10	600	9999	Fast cooling
SF11	600	9999	Fast cooling
BR1	600	9999	Fast cooling
BR2	600	9999	Fast cooling
PVD	-	-	-

Supplementary Table 2. Effective mass obtained by the LK-formula fitting of the T-dependent SdH oscillations in p-SnSe with different configurations of I and B.

Samples	Field direction	Current direction	Effective mass (m_e)
SF1	**B**//a-axis	**I**//c	0.341
		**I**//b	0.295
	**B**//b	**I**//c	0.259
	**B**//**c**	**I**//b	0.307
SF3	**B**//a	**I**//c	0.211
		**I**//b	0.194
SF6	**B**//a	**I**//bc plane	0.271
SF11	**B**//a	**I**//bc plane	0.368
SF12	**B**//a	**I**//bc plane	0.337
BR1	**B**//a	**I**//bc plane	0.227
BR2	**B**//a	**I**//bc plane	0.242

Supplementary Table 3. Statistics of point dislocations on randomly scanned areas of SnSe crystals. With a scanning size of $10 \times 10 \mu\mathrm{m}^2$, the average density is about $4.5 \times 10^4\,\mathrm{mm}^{-2}$. The scanning locations are randomly chosen, with a seperation larger than 1 mm, on freshely exfoliated SnSe surface.

Area Number	1	2	3	4	5	6
Number of dislocations	7	3	3	3	9	2
